# Gamification and transmedia in interdisciplinary contexts: A didactic intervention for the primary school classroom

**DOI:** 10.1016/j.heliyon.2021.e07374

**Published:** 2021-06-22

**Authors:** Mónica Ruiz-Bañuls, Isabel María Gómez-Trigueros, José Rovira-Collado, María Luisa Rico-Gómez

**Affiliations:** a Innovation and Didactic Training Studies Department, University of Alicante, Spain; b General and Specific Didactics Studies Department, University of Alicante, Spain

**Keywords:** Educational, Game, Information and communication technology (ICT), Student motivation, Transmedia storytelling

## Abstract

Previous research has established the lack of motivation by primary education students vis-à-vis the acquisition of the necessary key competencies compulsory in the Spanish school curriculum and this is closely linked to poor academic performance on the part of the aforesaid students. Based on this current educational reality, we present a quantitative study regarding the perception of students after the implementation of a new gamified experience in the classroom such that it integrates, for the first time and jointly, the benefits of gamification (Deterding et al., 2011a; Deterding & Zagal, 2018) with interdisciplinary methodologies (Candel, 2018; Cruz-Pichardo & Cabero-Almenara, 2020; Flores-Aguilar, 2019) and technology-enhanced transmedia narratives (Scolari 2018a, 2018b; Jenkins, 2003; Scolari 2018a, 2018b). This integrative and innovative proposal was implemented in a primary school in Valencia (Spain) between October 2019 and February 2020. The research is designed based on survey-type studies, employing an experimental cross-sectional quantitative methodology with pretest and posttest, in addition to experimental and control groups. Regarding the cross-sectional approach, descriptive analyses (means and standard deviation) have been conducted, together with histograms of each of the items in the questionnaire, as well as the *Student's t* test and an analysis of variance, which is of significant value when working with independent samples from a normal distribution, as in our case. The instrument utilised in the present study confirms the existence of a high and adequate internal consistency (Alpha = .873). The results obtained, as presented herein, confirm that the incorporation of the gamified proposals in primary school classes, unprecedentedly intertwined with the benefits provided by interdisciplinary work and transmedia narratives, notably improves the students' training process and their motivation, while also contributing to the better acquisition of compulsory curricular contents and enhanced academic performance.

## 1 Introduction and literature review

Gamification in the area of training and education continues to be challenging for teachers who are committed to interactive and participatory learning, thereby resorting to innovative methodologies wherein students are the true protagonists (Cruz-Pichardo and Cabero-Almenara, 2020; Gil-Quintana and Prieto-Jurado, 2019). As an emerging pedagogy, it provides active learning environments that integrate digital technologies (Adukaite et al., 2017; Contreras-Espinosa, 2016; Sosa et al., 2018; Trujillo, 2014; Trejo, 2019) aiming to build collaborative, creative, and innovative projects. This investigation is undertaken to analyse primary education students' perception of the process of developing significantly gamified and well-structured learning processes based on interdisciplinary and transmedia narrative axes (Fadeeva et al., 2010; Jenkins, 2003; Scolari, 2013). Likewise, we analyse the extent to which the students’ motivation increases (Buckley and Doyle, 2016) as a result of gamification-based pedagogical practices and how their implementation contributes to the acquisition of curricular content and training in key skills, especially towards improving the academic performance of students.

The incorporation of gamification into the educational domain (Burke, 2012; Deterding, 2012) is increasing, and this methodology has been receiving considerable attention and it is currently considered as one of the most useful and implementable resources in modern education (Ourachi et al., 2020; Parra-González et al., 2020; Wiggins, 2016). Although this term first emerged in 2008 (in a blog post by Brett Terrill), the concept did not become generalised until its use by Deterding, Dixon, Khaled, and Nacke in 2011, who defined it as ‘the use of game design elements in non-game contexts’ (2011a, p. 10). Kapp (2012a, 2012b) defines it as ‘using game-based mechanics, aesthetics, and game thinking to engage people, motivate action, promote learning, and solve problems’. Werbach and Hunter (2012) describe it as the use of game design elements applied in non-playful contexts, while Marín and Hierro (2013) coin a definition wherein gamification entails a simultaneous technique, method, and strategy to create meaningful and motivating experiences designed to achieve a common objective. Teixes (2015) also reports that the term can be defined as ‘the application of game resources (design, etc.) in non-playful contexts to modify the behaviours of individuals by acting upon their motivation to achieve specific objectives’ (2014, p. 23). As Cruz-Pichardo and Cabero-Almenara state, regardless of the definition and specificity assigned to the term ‘gamification’, ‘its strong association with games and play should not be forgotten, even if we can see that it has a much deeper background than simply playing, because rather, it is based on the idea of playing to learn’ (2020, p. 69).

In recent years, we have witnessed an explosion of the use of the term in specialised journals, presenting it as an important and new educational method as well as a highly attractive innovative instrument for 21st century students (Martí-Parreño et al., 2016; Ortiz-Colón et al., 2018; Parra-González and Segura-Robles, 2019; Peirats Chacón et al., 2019) not only in educational environments but also in a wider range of contexts (Deterding and Zagal, 2018; Martínez-Bravo et al., 2020; Shen et al., 2020; Patricio et al., 2020; Xi and Hamari, 2019). There is a considerable amount of gamification-related literature (Gómez and García, 2018), including how and when to introduce it into classrooms and the attendant benefits its use can have on teaching and learning processes. Notably, in recent years, the use of gamification practices has been described at various educational stages (Carrasco et al., 2019; Corchuelo-Rodríguez, 2018; Cruz-Pichardo and Cabero-Almenara, 2020; Chan et al., 2018; Deterding and Zagal, 2018; Fernández-Gaviria et al., 2018; Ferreira and Lacerda, 2018; Gil-Quintana and Prieto Jurado, 2019; Martínez-Franco, 2017; Parra-González et al., 2020; Pasalic et al., 2017; Ourachi et al., 2020; Pérez-López et al., 2017; Pisabarro and Vivaracho, 2018; Corchuelo-Rodríguez, 2018; Fernández-Gaviria et al., 2018; Ferreira & Lacerda, 2018; Pérez-López and Rivera-García, 2017; Quintero et al., 2018; Chan et al., 2018; Martínez-Franco, 2017).

According to extensive research, gamification tends to generate a high level of students’ commitment and involvement in subjects (Contreras-Espinosa, 2016; Hanus and Fox, 2015), as it facilitates intrinsic and extrinsic motivation (Buckley and Doyle, 2016; Conway, 2014; Hassan et al., 2019; Llopis and Balaguer, 2016; Sailer et al., 2017) and improves reflective thinking, collaboration, and problem solving skills (Aguilar and Aguilar, 2018; Cortizo et al., 2011).

The present study makes a major contribution to research on educational innovation by jointly encompassing three concepts of great relevance—gamification, interdisciplinarity, and transmedia narratives, while considering the current context, whereby gaming is increasingly present in our society and everyday lives as a form of leisure that we engage in different contexts and situations, with the aim of gaining pleasant and single experiences for ourselves and other people. The gamification concept and its operationalisation in non-gaming contexts have become a growing practice in several contexts (Deterding and Zagal, 2018; Patricio et al., 2020). Furthermore, its effectiveness in the primary education stage is analysed, unlike most of the similar proposals that have focussed on other stages, such as secondary (Hernández and Rovira-Collado, 2020), or on the teaching of foreign languages (Martín-Queralt and Batlle-Rodríguez, 2021; Rodrigues and Bidara, 2016).

Educative innovation (Pavitt, 2006) is understood in the literature as the pedagogical practice that is aimed at enhancing academic performance. Innovation needs not be related to the use of new technologies or methodologies, although quite often, they are directly related. Most of these new methodologies, such as gamification, i.e., the major focus of our research, do also exert an influence on students’ motivation (Buckley and Doyle, 2016; Hanus and Fox, 2015; Deterding, 2012). Our project is innovative as it focusses on increasing the motivation of primary education students and developing key competencies (EU, 2019), a major element within our curriculum (Tiana et al., 2011).

In view of the theoretical framework described above, we aim to address the following research objectives in the present study: (a) assess how gamification becomes a beneficial didactic strategy for the acquisition of curricular content which can significantly improve learning among students; (b) analyse the functionality of gamification for the development of interdisciplinary learning strategies with transmedia elements; (c) analyse the possibilities of the transmedia narratives in primary school; and (d) examine the level of student motivation towards academic performance after implementing a gamified proposal in the classroom that consequently promotes the improvement of the teaching–learning process itself. Based on a transversal methodology, our study explores the data obtained from all the students belonging to a whole educational level in a Spanish school.

The data obtained have been analysed from a descriptive (mean and standard deviation) and experiential perspective, through different analyses and processes (t-test, analysis of variance (ANOVA), and factorial analysis), from the configuration of an experimental and a control group. We hypothesise that the combination of the principles of gamification, interdisciplinarity, and transmedia narratives represents a didactic strategy with an immeasurable and innovative potential that has not been examined in previous publications, and which corresponds to the profile of the students we are targeting, i.e., users of multiple immersive platforms (Scolari, 2018a, 2018b) ‘who manage multiple tasks at high speeds and prefer to personalise and manage their own experiences, making them their own and actively participating in/creating them’ (Pérez-Manzano and Almela-Baeza, 2018, p. 96). We focus on primary education students, as they usually have less gamification experience, and show that this dynamic approach is completely feasible at this academic stage.

## 2 Research objectives

The present study is aimed at providing a new manner of approaching primary education such that students at this initial educational level acquire, through more motivating and innovative methodologies, the key competencies that must be fulfilled in their school curriculum. In this sense, based on gamified interdisciplinary proposals, new teaching strategies that pursue a comprehensive training of the students are introduced. These are perceived as motivating and highly inclined towards improving the students’ academic performance.

This investigation has been conceived from the following assumptions: the null hypothesis (H_0_) posits that classroom practice following a traditional teaching methodology hinders the acquisition of disciplinary contents and, therefore, the attainment of key competencies by the learners; while, the alternative hypothesis (H_1_) states that classroom practice with interdisciplinary gamified methodologies enables the acquisition of the contents within the field involved and, consequently, the achievement of the key competencies by that part of the learners.

Similarly, another objective is to promote the use of information and communication technologies (ICTs) in the classroom, as in the design of our proposal, the use of new technologies is essential to increase student motivation. As ICTs are used to access transmedia narratives, they serve as a support, among others, to the gamified proposal from the animated series *Avatar: The Last Airbender*. The benefits of the gamified strategy have been well established from the existing body of literature relating to the use of gamification (Kapp, 2012b; Lew et al., 2020; Parra-González et al., 2020; Pisabarro and Vivaracho, 2018). However, our research incorporates transmedia resources (Scolari, 2018a, 2018b) and interdisciplinary work (Candel, 2018; Cruz-Pichardo and Cabero-Almenara, 2020; Flores-Aguilar, 2019), implementing all these strategies together in the classroom for the first time. Our objective is to demonstrate how this comprehensive methodology is perceived by students not only as a new and useful procedure to achieve a rapid acquisition of the curricular content of the areas involved, as confirmed by the results obtained, but also as a helpful resource for the global training of key competencies, thereby increasing learners’ motivation towards studying in the primary education classroom, thus improving their academic performance.

## 3 Materials and methods

We use a quantitative, survey-based, cross-sectional pretest and posttest experimental methodological design with experimental groups (Groups A, B, F, and G) and control groups (Groups C, D, and E; Table 1). Analytical questionnaires are designed ad hoc to include the motivation components described by Álvarez Pérez et al. (1998), as well as to assess students' perception of gamified methodologies and their basic or key skills. Two different instruments are designed for the present study, whose content is validated by experts from Spanish public universities (Alicante, Murcia, the Balearic Islands, and Burgos) as well as foreign universities (in Portugal, Ecuador, and Cuba) following the ‘expert panel’ method: one for use prior to the didactic intervention and the other for post-intervention use. Such a method is based on the consultation with academics whose areas of specialisation focus on the theme of our investigation (all curricular areas, as described by the Spanish current regulations), as well as on the context wherein we aim to implemented the study (primary schools). The panel comprises experts (over 10 members) from different backgrounds (Universities of Burgos, Elche, Murcia, Salamanca, Albacete, and Baleares), to ensure complementary and diversified perspectives, who showcase their expertise in specific didactics and interdisciplinary gamified methodologies to help interpret the proposed intervention and the designed questionnaire. The appropriate composition of the body as well as the varied origins of its members in conjunction with their vast knowledge constitute essential features that aim to ensure the success of the foresight activity (Pinto, 2015; López-Gómez, 2018).

**Table 1:** Distribution of the participants by gender and group.

Group	Female	Age			Male	Age			Total
		9	10	11		10	11	12	
**Experimental Group**									
Group A	18	2	15	1	8	3	4	1	26
Group B	18	1	14	3	8	3	3	2	26
Group F	21	2	17	2	6	2	3	1	27
Group G	20	2	15	3	6	2	3	1	26
**Control Group**									
Group C	18	2	15	1	8	3	4	1	26
Group D	18	2	14	2	8	2	4	2	26
Group E	18	2	14	2	8	3	4	1	26

### 3.1 Ethics approval

The research has been conducted in compliance with the ethical standards required for research with human beings, respecting the basic principles included in the Helsinki Declaration and the code of good practice in research of the host university. The research has been approved by the management team of the educational centre (Centre Code: 03007352).

### 3.2 Instruments

Two questionnaires are used, one prior to the implementation of the gamified methodology with transmedia narratives, and the other thereafter. The pre-intervention instrument consists of 23 items measured on a five-point Likert scale (1, Strongly disagree to 5, Strongly agree), and it is organised into three study dimensions: 1. Sociodemographic characteristics (items 1–3); 2. Student motivation towards learning (items 4–13); and 3. Key skills (items 14–23). The post-intervention instrument comprises 33 items, which are also measured on a five-point Likert scale and structured into four analysis groups: 1. Sociodemographic characteristics (items 1–3); 2. Student motivation towards learning (items 4–13); 3. Key skills (items 14–23); and 4. Perception of gamified methodologies and transmedia narratives (24–29). To verify the reliability of the questionnaire, we calculate the Cronbach alpha coefficient to obtain α = 0.873, which confirms that the proposed instrument has a high internal consistency that is adequate for the present work (Bisquerra, 2004; Hernández et al., 2003). It is noteworthy that the questionnaire is submitted in a guided and semi-structured manner by the educators. The complexity of the plausible questions is framed within the context emerging between the tutor's mediator role and the complex expository reading process (García, 2014; Sainz, 2005). Adopting mediated reading as a metacognitive strategy tackles possible reading comprehension gaps among the students who present difficulties in the formulation of the questions.

### 3.3 Participants

A total of 183 students in their fifth year of primary education at a Spanish public centre participate in this research. These children are aged between 9 and 11 years and represent all the students in that school year (N = 183). We organise them into seven groups, ranging from A to G. Their distribution by gender is 52 males (28.6%) and 131 females (71.4%) (Table 1).

The experimental group consists of a cohort of 105 students who work on the contents through the gamified, transmedia, and interdisciplinary project. The control group comprises 78 students who work on the same contents of each of the areas involved with a traditional methodology (Engeström et al., 1999; Eklund and Dowdy, 2014) based on the use of the textbook. The selection of participants is non-probabilistic and incidental (Bisquerra, 2004). Natural groups are maintained, leaving the participants in their class groups.

### 3.4 Process

We follow a usual experimental research strategy, with control and experimental groups. Thus, the pretest questionnaire is administered to the entire sample (N = 183) through a link hosted on the free-to-use Google Forms application before the gamified intervention is carried out in the classroom. As the participants are minors aged between 9 and 11 years old, their legal guardians are informed of the objective of the study and the confidentiality with which the collected data would be treated through a letter distributed by the Centre to request their consent for the investigative use of the responses obtained. The instrument was implemented in October 2019, and subsequently, the didactic activities were carried out in the experimental groups throughout December (2019) and January (2020).

In the present study, we aim to test the implementation of a project that would allow learning the curricular content using a well-structured interdisciplinary gamified strategy (Ortega-Sánchez and Gómez-Trigueros, 2019) based on the use of transmedia narratives (Scolari, 2013). Gamified proposals always include a concrete narrative that enables the comprehension of the story of the project beyond literary references (Núñez et al., 2019). Transmedia narratives offer multiple gamification approaches, from books to videogames (Vizcaíno-Verdú, Contreras-Pulido and Guzmán-Franco, 2018). Although the possibilities of working on literary education through videogames have already been contrasted (Serna-Rodrigo and Rovira-Collado, 2021), we discover that several other interdisciplinary proposals expand the possibilities of these narratives (Scolari, 2018b).

All the materials we develop are based on the animated series—Avatar: The Last Airbender—which is a narrative universe well known to the students targeted by this intervention and is associated with several multimodal elements (Kress, 2010), including television series, graphic novels, movies, comics, video games, and short novels, of a complete transmedia universe. The main plot of the series follows the journey of Aang and his friends through their universe to master the elements of air, water, earth, and fire. In this intervention, we associate these elements with four of the students’ elementary curriculum subjects: Spanish language, mathematics, natural sciences, and social sciences, as well as with their corresponding key skills.

By focussing on a consolidated transmedia narrative with multiple extensions on different platforms, our gamification approach can be considered as another extension for educational purposes. In the first session, we look at the first chapter of the *Avatar* series, explain the working methodology, and explore the project website which has different spaces that contain the system resources, including collectible cards, experience bars, context stories for each clan, project photographs, announcements, the complete didactic sequences, a list of rewards, and a list of missions. Each group invents a clan name, designs its logo and emblem using the Cool Text and Makebadges virtual tools, and contextualises its clans within the narrative universe of Avatar by writing a description that is included in the digital platform. Small heterogeneous groups are formed with six to seven members, and these constitute the clans in the fictional universe of the series. Many of the activities in the proposal are designed to help the entire team achieve common goals, thereby promoting cooperative learning (González and Carrillo, 2016; Sánchez et al., 2010).

In designing our proposal, the use of ICTs is essential, as they are used to access transmedia narratives and serve as a support, albeit not the only one, for the gamified proposal (Peirats-Chacón et al., 2019). Although the activity is carried out prior to the coronavirus disease 2019 (COVID-19) outbreak, we could establish that the use of ICTs has already been unavoidable in primary schools. Although some challenges and difficulties in their use in public schools are still identifiable (Salam et al., 2018), there are numerous factors (Lim, 2015) that guide their use in didactic proposals.

The main mechanics used are experience points, a level progression system, badges, missions, as well as both virtual and physical item rewards (Borrás-Gené, 2018; Gil-Quintana and Prieto Jurado, 2019; Werbach and Hunter, 2012). The students have had user profiles on the Internet where they could check their progress in the different curricular areas which are represented with circular experience bars that are completed as they perform the required tasks and optional missions. Most of the tasks are interdisciplinary, and experience points are obtained through five tests wherein the students have to use the skills and knowledge they have acquired in different academic disciplines.

Finally, the posttest questionnaire was completed by all the participants in February 2020, fortunately before the COVID-19 confinement was decreed. We conduct descriptive analyses by calculating the means and standard deviations for the data, using the SPSS software (version 23 for Windows, IBM Corp., Armonk, NY). The normality of all the data sets is verified by analysing histograms and QQ graphs for each of the questionnaire items. Similarly, we conduct a Kolmogorov–Smirnov test, and find significance levels exceeding a probability of 0.05 (p < 0.05), thus confirming the normal distribution of the data (Argibay, 2009). In our subsequent analyses, we apply Student's t-tests and ANOVA tests for normally distributed independent samples (Pardo & Ruiz, 2002).

The statistics presented respond to the objectives of this research. On the one hand, using descriptive statistics, we manage to verify the extent to which the students are motivated towards studying before and after the gamified intervention, with transmedia and interdisciplinarity, to assess the relevance of the proposal, and whether or not it is a beneficial didactic strategy for the acquisition of curricular content.

On the other hand, statistics permit the evaluation of the key competencies of the participants before and after the intervention (Table 2); Student's t statistical tests and ANOVA allow us to analyse the existence of significant differences between the groups (experimental and control) in the variables studied (Table 3). The descriptive analyses results presented in Table 4 are evaluations of the perception of the gamified experience with the transmedia narratives of the experimental students.

**Table 2:** Descriptive results obtained for the pre- and post-intervention instruments.

^1^D.	Item	Experimental *g*roup				Control group			
		Pre		Post		Pre		Post	
		^2^M	^3^SD	^2^M	^3^SD	^2^M	^3^SD	^2^M	^3^SD
Motivation to study	Item 4	2.89	0.997	2.08	0.409	2.97	0.994	2.97	0.998
	Item 5	2.43	0.999	4.41	0.432	2.42	0.966	2.47	0.961
	Item 6	2.69	1.080	4.55	0.378	2.76	0.951	2.79	0.992
	Item 7	2.36	0.965	1.90	0.338	2.30	0.912	3.45	0.904
	Item 8	2.68	0.983	4.64	0.033	2.89	0.979	2.87	0.983
	Item 9	4.77	0.998	2.81	0.369	4.73	0.982	3.79	0.997
	Item 10	2.41	0.920	1.90	0.362	2.31	0.912	3.35	0.914
	Item 11	2.67	0.996	4.80	0.321	2.78	0.989	2.78	0.996
	Item 12	2.69	0.989	4.78	0.354	2.72	0.973	2.77	0.992
	Item 13	4.32	0.977	1.95	0.325	4.35	0.904	4.36	0.907
Key competencies	Item 14	2.38	0.563	4.71	0.461	2.15	0.419	2.18	0.416
	Item 15	2.33	0.551	4.83	0.417	2.18	0.412	2.19	0.418
	Item 16	2.40	0.533	4.88	0.335	2.19	0.461	2.20	0.419
	Item 17	4.19	0.954	4.97	0.324	4.09	0.930	4.11	0.941
	Item 18	2.39	0.570	4.87	0.316	2.11	0.423	2.18	0.424
	Item 19	2.13	0.542	4.91	0.321	2.01	0.516	3.09	0.419
	Item 20	4.21	0.976	4.85	0.314	4.04	0.943	4.09	0.946
	Item 21	4.12	0.994	4.86	0.317	4.06	0.887	4.11	0.883
	Item 22	2.39	0.601	4.81	0.322	2.12	0.419	2.18	0.417
	Item 23	4.19	0.967	4.92	0.308	4.17	0.941	4.03	0.942

Item
4. If the teacher asks volunteers to correct in the whiteboard, I would only participate if I am sure I can do my best.
5. Although the teacher does not give me homework, I like to spend time studying daily.
6. For me, it is important to get a good result and to also know that I am among the best.
7. I don't care if other students are more hardworking than I am.
8. If a friend gets a better result than I, I want to study and do better than he or she.
9. After doing a test I am usually stressed out until I get my mark.
10. When I get bad results, I usually think that it's not just my fault.
11. What I'm learning in the lesson is more important than the mark I am getting in the exam
12. Relating the contents of some subjects to others would help me understand them better.
13. Before starting a difficult exercise, I often think that I will not do it well.
14. I am able to express and interpret concepts, thoughts, feelings, facts, and opinions orally and in writing (listening, speaking, reading, and writing) in an appropriate and creative way.
15. I have improved my communication in foreign languages, and I am able to understand, express myself, and interpret concepts, thoughts, feelings, facts, and opinions orally and in writing (listening, speaking, reading, and writing).
16. I can develop and apply mathematical reasoning to solve different problems in everyday situations through the mastery of calculation.
17. I am knowledgeable in the use of computers and tablets, and I can use these resources to work and learn both individually and collectively.
18. I am able to gain, process, and assimilate new knowledge and skills, as well as look for guidance and use it to learn.
19. I have the ability to participate effectively and constructively in the social life of the classroom and school, and I am also able to resolve conflicts that may arise in teamwork.
20. I consider that I have the ability to transform ideas into actions, while planning and managing my creativity to achieve my goals.
21. I can express my ideas, experiences, and emotions through different media, including music, performing arts, literature, and arts and crafts.
22. I am able to search for information, process it, and then use it for my work and further learning.
23. I have the ability to use different resources and sources to organise my ideas and use them, both collectively and individually.

D. = dimension; ^2^M = mean; ^3^SD = standard deviation.

**Table 3:** T-tests and one-factor analysis of variance (ANOVA) for the experimental and control groups.

Item		Student's t-test		ANOVA	
		t	p	F	p
Motivation to study	Item 4	-6.898	0.071	83.171	0.081
	Item 5	-9.174	0.000∗∗	117.334	0.001∗∗
	Item 6	-10.940	0.003∗∗	105.444	0.002∗∗
	Item 7	-13.803	0.002∗∗	108.706	0.004∗∗
	Item 8	12.444	0.090	67.429	0.090
	Item 9	10.406	0.092	47.531	0.107
	Item 10	-13.803	0.000∗∗	99.893	0.004∗∗
	Item 11	-12.658	0.000∗∗	102.449	0.001∗∗
	Item 12	-12.315	0.000∗∗	112.496	0.002∗∗
	Item 13	-11.938	0.064	64.815	0.121
Key competencies	Item 14	25.460	0.002∗∗	101.083	0.003∗∗
	Item 15	23.384	0.003∗∗	113.214	0.002∗∗
	Item 16	21.663	0.034	49.129	0.089
	Item 17	2.178	0.021	43.837	0.103
	Item 18	24.503	0.001∗∗	133.244	0.001∗∗
	Item 19	25.112	0.039	50.211	0.137
	Item 20	2.031	0.003∗∗	120.003	0.002∗∗
	Item 21	2.452	0.008∗∗	91.837	0.006∗
	Item 22	26.878	0.000∗∗	112.456	0.001∗∗
	Item 23	2.431	0.023	103.120	0.018∗

According to the Levene statistic, the variances were equal for all the outcomes (p > 0.05) ∗p < 0.05; ∗∗p < 0.01.

**Table 4:** Descriptive results for the gamified intervention with transmedia narratives proposal. Experimental group.

Item	Experimental group	
	^1^M	^2^SD
Item 24. Group work motivates me to keep learning.	4.86	0.413
Item 25. Getting points and other items in the game encourages me to work.	4.82	0.351
Item 26. I really liked going on missions for all of the subjects.	4.75	0.378
Item 27. Learning through playing with transmedia narratives is a lot of fun for me, and it motivates me to keep learning.	4.95	0.331
Item 28. I try harder and motivate myself to earn experience points and level up.	4.94	0.341
Item 29. This way of learning was more interesting to me and encouraged me to study more.	4.96	0.368
Item 30. The use of comics, television series, and different platforms helped me to learn more easily.	4.91	0.344
Item 31. The varied resources, other than textbooks, encouraged me to actively participate in my learning.	4.92	0.351
Item 32. I was encouraged to complete the missions I saw in resources such as videos or on the Moodle platform.	4.83	0.339
Item 33. Using missions and transmedia to learn about new content allowed me to learn faster.	4.80	0.391

^1^M = mean; ^2^SD = standard deviation.

All these analyses allow us to reach conclusions about the relevance of this type of study, ensuing a highly significant learning outcome for the students. Likewise, they enable the positive assessment of the functionality of gamification for the development of interdisciplinary learning with transmedia elements and, finally, to examine the degree of motivation of students linked to academic performance after implementing the gamified proposal in the classroom, which consequently promotes the improvement of teaching–learning process.

## 4 Results

### 4.1 Descriptive analysis

Table 2 shows the values obtained for the descriptive statistics for both the pre- and post-intervention instruments. These results indicate that student motivation in the phase prior to the classroom intervention with gamified methodologies is negative. Both the experimental and control student groups show scores for the study time variable (item 5, M ≥ 2.43) and the test marks they might obtain (item 7, M ≥ 2.30; item 10, M ≥ 2.31), with mean values of around two points (Disagree) and a high pre-intervention response dispersion (SD ≤ 0.965).

Likewise, the students presented unfavourable attitudes towards the academic results they might obtain in their tests, with average scores close to five points (item 9, M ≥ 4.73; SD ≤ 0.998). Similarly, negative results are obtained when they are asked about their motivational self-esteem towards solving the exercises that are set (item 13, M ≥ 4.32, SD ≤ 0.977). They also place little importance on learning content in the classroom (item 11, M ≥ 2.67, SD ≤ 0.996) or on the value of interdisciplinary learning (item 12, M ≥ 2.69, SD ≤ 0.989) to acquire new interrelated knowledge.

Regarding key skills, the sample notably has a positive perception of becoming familiar with digital skills and the use of transmedia narratives, obtaining scores exceeding four points in both cases (item 17, M ≥ 4.09; item 23, M ≥ 4.17), with deviations in both the experimental and control groups exceeding 0.9 points (item 17, SD ≤ 0.954; item 23, SD ≤ 0.967). Similarly, the students respond positively when asked about their training in entrepreneurial spirit and sense of initiative (item 20, M ≥ 4.04; item 21, M ≥ 4.06). The same does not occur with the other skills: linguistics (item 14, M ≥ 2.15; item 15, M ≥ 2.18); mathematics (item 16, M ≥ 2.19); learning to learn (item 18, M ≥ 2.11); social and civic skills (item 19, M ≥ 2.01); and cultural expression (item 22, M ≥ 2.12), with average response values of two points.

After the implementation of this gamified didactic proposal with transmedia narratives, the results change substantially in the experimental group compared to the control group. Thus, a significant increase in student motivation to work is confirmed in the experimental group (item 5, M = 4.41; item 6, M = 4.55) with a considerably reduced standard deviation (item 5, SD = 0.432; item 6, SD = 0.378), thereby suggesting that there is a generalised change in attitude in the experimental group students. Similarly, the students in the experimental group place greater importance on obtaining good grades (item 7, M = 1.90; item 10, M = 1.95), with average response values close to two points (item 7, SD = 0.338; item 10, SD = 0.362).

Likewise, the students also show a proactive attitude towards their academic results by completing extra activities and exams after participating in the gamified intervention with transmedia elements, with responses averaging two points (item 9, M = 2.81; SD = 0.369). Positive results are also obtained for motivational self-esteem towards solving academic exercises in the experimental group (item 13, M = 1.95, SD = 0.325) compared to the control group, whose results remain negative (item 13, M = 4.36, SD = 0.907), thus further suggesting the benefits of these classroom strategies.

In relation to the importance of learning in class, in the experimental group there are increased scores assigned to the work carried out in the classroom (item 11, M = 4.80, SD = 0.321) and for the variable of interdisciplinary learning with transmedia narratives to acquire new interrelated knowledge (item 12, M = 4.78, SD = 0.354). Finally, after the gamified intervention, the students report a positive perception of the training in key skills, with results close to the maximum response value of five points (items 14–23, M ≤ 4.97) and an SD lower than 0.5 (items 14–23, SD ≥ 0.308) for all the items (Table 2).

### 4.2 Comparative analysis of the experimental versus control groups

To evaluate the didactic and motivational functionality of the use of a well-structured gamification strategy based on interdisciplinary, transmedia narratives in a primary education classroom, we compare the results obtained in the experimental groups (Groups A, B, F, and G) with the control groups (Groups C, D, and E) using Student's t-tests for independent samples. Our results (Table 3) indicate that there are significant differences between the groups for the variables studied.

The confidence interval limits for the differences indicate that the value 0 is not included in any of the variables, meaning that the hypothesis of equality of the means can be rejected for these items, as confirmed in the t-test results for these variables. The results returned in the analysis of variance show that there are significant differences between the experimental and control groups for the dimensions analysed (p < 0.01). Thus, the participants in the experimental group place greater importance on the variables related to motivation towards study than the students in the control group (item 5, p = 0.001; item 7, p = 0.004). The results are similar for the variables related to responsibility and personal work (item 6, p = 0.002; item 10, p = 0.004), which the students positively valued in the experimental group, with an average value of approximately five points (Totally agree) compared to lower scores in the control group students.

Furthermore, in relation to the control group, the students in the experimental group consider that learning content, regardless of the grades they obtain in the tests (item 11, p = 0.001), and the acquisition of new knowledge as a result of interdisciplinary work with transmedia narratives in class are important (item 12, p = 0.002). In general, significant differences are found for these variables between the participants in the experimental versus the control groups (Groups A, B, F, and G versus Groups C, D, and E), with higher mean values obtained for all the variables in the experimental group.

Similarly, there is an improvement in the experimental group compared to the control group in the self-assessment scores for all the questions related to the key skills dimension. In particular, the scores for the perception of competence in linguistic communication (item 14, p = 0.003; item 15, p = 0.002), learning to learn (item 18, p = 0.001), sense of initiative and entrepreneurial spirit (item 20, p = 0.002; item 21, p = 0.006), and cultural expression (item 22, p = 0.001) are noticeable for their notable improvements.

### 4.3 Analysis of the perception of the gamified experience with transmedia narratives

As shown in Table 4, the students in the experimental group receive the interdisciplinary transmedia gamified experience methodology well, and the interdisciplinary learning they acquire is particularly noteworthy. First, these data indicate that game-based experiences promote the development of team and collaborative work, as shown by the answers to item 24 (M = 4.86; SD = 0.413), which are close to five points (Totally agree). Second, there is an increase in student motivation for the completion of work in the classroom (item 25, M = 4.82; SD = 0.351), comprehensive study (item 29, M = 4.96; SD = 0. 368), and interest in learning (item 27, M = 4.95; SD = 0.331).

**Figure 1:**
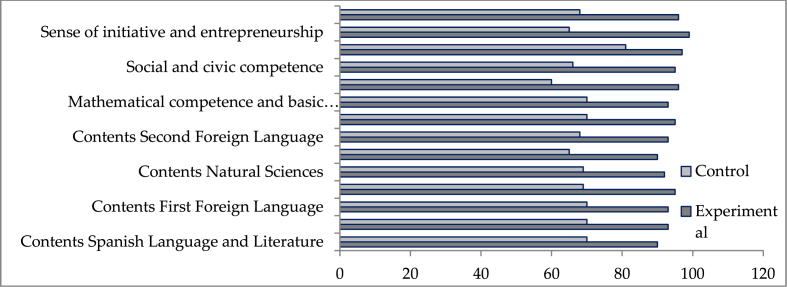
Results of the final tests after the gamified intervention in the experimental and control groups.

Finally, it should be noted that all these descriptive values are corroborated by the results obtained in the tests we conduct (Figure 1).

The compulsory key competencies to be developed according to the primary school curriculum, as well as the didactic areas analysed in this interdisciplinary study (Spanish Language and Literature; Social Science) show better results among the students of the experimental group in all the learning dimensions analysed (contents and competencies).

Similarly, results close to 97% confirm the achievement of the initial educational objectives of other areas involved in the interdisciplinary gamified proposal with transmedia narratives (contents of the following subjects: Mathematics; First Foreign Language; Natural Sciences; Art Education; and Second Language Foreign) as well as the achievement of the contents of the areas of Spanish Language and Literature, and Social Sciences.

Furthermore, the training in key competencies shows positive results in the experimental group, with values that exceed 95% in their evaluation, while in the control group they do not reach 70%.

## 5 Discussion

In this section, we discuss the results and how they relate to the research questions we address in the present work. Our first objective is to assess whether educational dynamics gamified through transmedia narratives improve the motivation of students in their fifth year of primary education. There is a substantial improvement in the descriptive results (M and SD) obtained in student perception in the experimental group after the classroom intervention (post-intervention results). Likewise, Student's t-tests for independent samples in this same dimension show that there are significant differences between the treatment and the control groups, indicating that both the gamification and implementation of transmedia narratives effectively improve the students' motivation to study.

Similarly, these data also highlight the benefits of this type of methodology to promote work both inside and outside the classroom. All of these agree with the claims by various authors (Almeida and Simoes, 2019; Aguilar and Aguilar, 2018; Buckley and Doyle, 2016; Carrasco et al., 2019; Sailer et al., 2017; Palazón, 2015) that didactic proposals mediated through play activate students’ intrinsic motivations based on the simple pleasure of playing, thereby allowing students in educational contexts to discard the notion of traditional compulsory activities that are driven by extrinsic motivations. As demonstrated in the current research, the implementation of certain elements of games, such as levels, points, and badges, increases the time that the participants spend on the learning activity and helps to improve their predisposition to continue.

The promotion of teamwork is another advantage regarding the use of gamification in teaching. The second objective of the present study is to identify the influence of collaborative tasks through the students' reported sense of belonging to a group. When people feel that they belong to a group, their need for connection is satisfied (Cortizo et al., 2011; Hamari, 2013). As shown in the descriptive results and in the data obtained from the Student's t-tests, the students value teamwork very positively in the post-intervention phase of this research, thus confirming its function in motivating individuals to complete joint activities, thereby helping them to develop a sense of belonging to a group that shares a common goal. These conclusions converge with those obtained by Huang et al. (2018), Lew et al. (2020), Ortega-Sánchez and Gómez-Trigueros (2019), Parra-González et al. (2020), and Patricio et al. (2020), indicating that deep, positive feelings develop when experiences are shared, as evident in gamified context-induced learning, such as in the current research. These dynamics tend to create stronger bonds, leading to engagement, enjoyment, and increased motivation.

From the constructivist sphere, other educational possibilities offered by gamification entail the progressive construction of learning by students as they interact with novel, rich, and diverse environments (Contreras-Espinosa and Eguia, 2016; Zubiría and Doris, 2004). This progress is compounded by the development of commitment and autonomous (Perrotta et al., 2013) skills by overcoming challenges (Albrecht, 2014; Conway, 2014; Corchuelo-Rodríguez, 2018; Ourachi et al., 2020), as well as the acquisition of training, increased thinking, and coordination skills (Blanton, 2012). Proposals involving play and transmedia technology as well as narratives promote a new literacy (Scolari, 2018b) and favour the stimulation of certain affective factors, such as improved interactions between students, and the development of enhanced social skills (Pérez-Manzano and Almela-Baeza, 2018).

The third objective in the present study is to analyse the functionality of gamification in the development of interdisciplinary learning. In this sense, gamified actions in education are associated with immersive training experiences (Candel, 2018; Cruz-Pichardo and Cabero-Almenara, 2020; Flores-Aguilar, 2019) which aim to help students acquire a broader and deeper knowledge of the contents they are working on, mediated by the fun they experience by playing the game. Our analysis of the evaluation results and percentages of curricular content acquired by students in the control and experimental groups in different learning areas corroborates the idea that the implementation of gamification enhances learning and achieves better training performance (Haruna et al., 2018; Ortiz-Colón et al., 2018), while increasing student commitment to learning in the educational contexts wherein it is implemented. From this perspective, this methodology enriches interdisciplinary learning because it actively involves students and facilitates content interaction from different areas.

Another objective of the present study is to assess the usefulness of game mechanics and dynamics aimed at training in curricular content and key skills. Indeed, the values we obtain in the ANOVA test confirm that students in the experimental group acquire different types of skills (communicative, mathematical, social, cultural, and technological) as well as abilities in other cognitive processes, including empathy and creative thinking, when troubleshooting. Thus, the results we obtain suggest that games and gamification processes are useful and valid strategies for teaching and learning key skills in the primary educational stage, and that these skills can be used to help students to acquire knowledge in different content areas. These findings concur with those obtained by other authors (Ayén, 2017; Contreras-Espinosa and Eguia, 2016; Español, 2017) who suggest that these strategies can reinforce the positive attitudes of students, thereby enabling them to generate critical thoughts and procedural skills which facilitate their continued learning and help them to acquire new and complex knowledge. Likewise, our results reveal that the use of game dynamics in teaching–learning processes improves the ability of students to understand the meaning of new information, ask questions, make decisions, and draw conclusions that will help them to achieve expected learning objectives and outcomes (Hamari and Koivisto, 2015). The diagnostic nature of the results of this research could help to promote the implementation of the specific teaching activities designed to exploit gamification processes in the didactic treatment of the contents of primary education curricula.

## 6 Conclusions

Education, influenced by social transformation factors, plays a fundamental role in a societal context of global changes at the socioeconomic, cultural, political, technological, and demographic levels. Recent studies have shown that there is a decline of those traditional methodologies which rely largely on the memorisation of disconnected concepts and whose content is organised into separate disciplinary shells (Buckley and Doyle, 2016; Carrasco et al., 2019; Chan et al., 2018). Similarly, the didactic proposal analysed herein appears to highlight the advantages of implementing a game-based curriculum in formal educational contexts (Chan et al., 2018; Fleischmann and Ariel, 2016; Haruna et al., 2018). Indeed, the educational potential of playful simulation or gamified contexts and their connection with the real world has served as a foundation for several studies and proposals relating to the use of games (and video games) in educational settings (Corchuelo-Rodríguez, 2018; Fernández-Gavira et al., 2018; Holguin et al., 2020; Palazón, 2015; Serna-Rodrigo, 2020). Based on this approach, the link between the promotion of activities and learning (Adukaite et al., 2017; Yunyongying, 2014) and problem solving is alluded to by the learning achieved by these students based on curricular content about social problems delivered with gamified strategies and resources.

Motivation is one of the most acclaimed benefits of gamified methodologies (Flores-Aguilar, 2019; Mikel, 2016), and its potential is also confirmed in this investigation. Some authors claim that this methodology is successful mainly because it is based on the concept of ‘fun’ in relation to games (Hamari and Koivisto, 2015). However, this ignores the underlying fact that gamification represents a mechanism to increase student effort. This position, which is confirmed by the student participants in the present study, corroborates the intrinsic motivating value of gamified ICT-based interdisciplinarity activities which acts as a ‘springboard’ for participants who manage to recognise the true objectives of these proposed recreational activities (Almeida and Simoes, 2019).

Furthermore, the value of educational gamification is associated with the immersive training experiences it provides (Castellón and Jaramillo, 2013; Candel, 2018; Trejo, 2019), which can be considered as training activities that aim to help to students acquire a broader and deeper knowledge of the contents they are working on via the conduit of the fun of the game. Therefore, the implementation of gamification enhances learning, achieves better training performance (Haruna et al., 2018; Parra-González et al., 2020), and increases student commitment to learning in the educational contexts wherein it is implemented.

From this perspective, this methodology enriches interdisciplinary learning because it actively involves students, promotes cooperative learning (Gallego-Durán et al., 2014; Zarzycka-Piskorz, 2016), teamwork (García-Valcárcel et al., 2014; Gómez and García, 2018; Johnson et al., 2013), individual and collective decision-making, collaborative attitudes and behaviours (Ferreira and Lacerda, 2018; Ortiz-Colón et al., 2018), and favours the development of dynamics focussed on modifying certain negative behaviours, thereby generating healthy social habits (Borrás-Gené, 2017; Holguin et al., 2020) via the adoption of a methodological approach defined by the students who participate in this research as ‘the completion of challenges’. Accordingly, the sense of healthy competition between students to obtain group or individual points, tolerance towards making mistakes as a natural part of learning, curiosity, and experiential learning simulated by the didactic proposal or project would discourage students from developing a sense of apathy towards learning and acquisition of knowledge that teachers wish to impart as part of the curriculum.

Therefore, the present research focusses on the motivating potential of games and confirms that one of its positive characteristics is that it allows students to attain points, which encourages them to work in the classroom and progress in their learning. Likewise, the relevance of gamified dynamics can be regarded as the development of student motivation to carry out tasks in search of recognition by their classmates and teachers. These conclusions coincide with those reported in very recent works by Parra-González et al. (2020), Mahmud et al. (2020), and Carrasco et al. (2019), suggesting that competitive gamification activities serve to promote individual and group work by students, increase student participation and interaction, and construct support networks which intrinsically encourage collaborative work.

Summarily, the cross-sectional and descriptive analyses carried out in this research have verified that, given the current demotivation of primary education students in the process of acquiring key competencies that are mandatory in the Spanish school curriculum, the implementation of our didactic proposal has been perceived by students to be very positive in their training process. Our scheme has brought together, for the first time, a gamified intervention coupled with the benefits provided by an interdisciplinary methodology and the ICT innovation granted by transmedia narratives. Our proposal has notably increased students’ motivation, while similarly contributing to the better acquisition of compulsory curricular content and significantly increasing their academic performance.

## Declarations

### Author contribution statement

Mónica Ruiz-Bañuls & José Rovira-Collado: Conceived and designed the experiments; Performed the experiments; Wrote the paper.

Isabel María Gómez-Trigueros & María Luisa Rico-Gómez: Analyzed and interpreted the data; Wrote the paper.

### Funding statement

This research did not receive any specific grant from funding agencies in the public, commercial, or not-for-profit sectors.

### Data availability statement

Data included in article/supp. material/referenced in article.

### Declaration of interests statement

The authors declare no conflict of interest.

### Additional information

No additional information is available for this paper.
